# Adjuvant Use of the Invariant-Natural-Killer-T-Cell Agonist α-Galactosylceramide Leads to Vaccine-Associated Enhanced Respiratory Disease in Influenza-Vaccinated Pigs

**DOI:** 10.3390/vaccines12091068

**Published:** 2024-09-18

**Authors:** Bianca L. Artiaga, Daniel Madden, Taeyong Kwon, Chester McDowell, Cassidy Keating, Velmurugan Balaraman, Darling Melany de Carvahlo Madrid, Laurie Touchard, Jamie Henningson, Philip Meade, Florian Krammer, Igor Morozov, Juergen A. Richt, John P. Driver

**Affiliations:** 1 Department of Diagnostic Medicine & Pathobiology, College of Veterinary Medicine, Kansas State University, Manhattan, KS 66506, USA; 2 Division of Animal Sciences, University of Missouri, Columbia, MO 65211, USA; 3 Bond Life Sciences Center, University of Missouri, Columbia, MO 65211, USA; 4 Center for Vaccine Research and Pandemic Preparedness (C-VaRPP), Icahn School of Medicine at Mount Sinai, New York, NY 10029, USA; 5 Department of Pathology, Molecular and Cell-Based Medicine, Icahn School of Medicine at Mount Sinai, New York, NY 10029, USA; 6 Ignaz Semmelweis Institute, Interuniversity Institute for Infection Research, Medical University of Vienna, 1090 Vienna, Austria

**Keywords:** natural killer T cell, influenza A virus, vaccine, adjuvants, vaccine-associated enhanced respiratory disease, α-galactosylceramide, swine

## Abstract

Invariant natural killer T (iNKT) cells are glycolipid-reactive T cells with potent immunoregulatory properties. iNKT cells activated with the marine-sponge-derived glycolipid, α-galactosylceramide (αGC), provide a universal source of T-cell help that has shown considerable promise for a wide array of therapeutic applications. This includes harnessing iNKT-cell-mediated immune responses to adjuvant whole inactivated influenza virus (WIV) vaccines. An important concern with WIV vaccines is that under certain circumstances, they are capable of triggering vaccine-associated enhanced respiratory disease (VAERD). This immunopathological phenomenon can arise after immunization with an oil-in-water (OIW) adjuvanted WIV vaccine, followed by infection with a hemagglutinin and neuraminidase mismatched challenge virus. This elicits antibodies (Abs) that bind immunodominant epitopes in the HA2 region of the heterologous virus, which purportedly causes enhanced virus fusion activity to the host cell and increased infection. Here, we show that αGC can induce severe VAERD in pigs. However, instead of stimulating high concentrations of HA2 Abs, αGC elicits high concentrations of interferon (IFN)-γ-secreting cells both in the lungs and systemically. Additionally, we found that VAERD mediated by iNKT cells results in distinct cytokine profiles and altered adaptation of the challenge virus following infection compared to an OIW adjuvant. Overall, these results provide a cautionary note about considering the formulation of WIV vaccines with iNKT-cell agonists as a potential strategy to modulate antigen-specific immunity.

## 1. Introduction

Antiviral host responses involve complex interactions among multiple cell types and signaling pathways. Under certain circumstances, vaccination or viral infection induces detrimental immune responses that result in enhanced disease rather than protection after a subsequent infection. This phenomenon is often referred to as vaccine-associated enhanced disease (VAED). Some forms of VAED are associated with the presence of virus-specific non-neutralizing or sub-neutralizing antibodies (Abs) that facilitate virus uptake into the target cell, potentially via Fcγ-receptor or complement-dependent mechanisms [1,2]. VAED has been reported for a wide range of viruses including flaviviruses (Dengue, Zika, Murry Valley encephalitis virus, Japanese encephalitis virus, West Nile virus), respiratory syncytial virus, and many others (reviewed in [3]). It has also been demonstrated for influenza virus infections following immunization with an inactivated vaccine containing a virus of the same hemagglutinin subtype as the subsequent challenge strain, but with a substantial antigenic shift [4,5,6,7]. Influenza-mediated VAED has been demonstrated in several animal models including rabbits, pigs, and ferrets, as well as in cell cultures [4,5,6,7,8,9,10]. Furthermore, findings from preserved lower-airway tissues from fatal cases during the 1957 H2N2 pandemic as well as observations made during the 2009 swine influenza pandemic present the possibility that humans are also subject to this phenomenon [11,12,13].

A model of influenza-induced VAED, characterized by prolonged fever, severe pneumonia, bronchiolitis, interlobular and alveolar edema, hemorrhage, and elevated proinflammatory cytokines, has been demonstrated in pigs and ferrets [8,14]. Known as vaccine-associated enhanced respiratory disease (VAERD), this syndrome can be induced by vaccination with a monovalent whole inactivated virus (WIV), or recombinant HA subunits followed by a heterologous challenge with a virus expressing the same hemagglutinin (HA) subtype. Furthermore, the extent of immunopathology varies according to age and the different IAV strains and adjuvants used [6,7,15,16,17,18,19]. The mechanisms underlying VAERD are not fully understood. However, it has been associated with the presence of non-neutralizing Abs targeting a conserved region of the HA2 stalk, which are thought to enhance the membrane fusion activity of the virus, perhaps resulting in increased virus replication and pathogenicity [14].

VAERD induction requires that mismatched vaccines be administered with an adjuvant to elicit disease. So far, this has been demonstrated with oil-in-water (OIW) and squalene-based nano-emulsion adjuvants that are widely used in WIV swine vaccine formulations to amplify cross-reactive immune responses against heterologous IAVs [15]. These classes of adjuvants improve antigen release, stability, and uptake and/or they use broadly specific immune stimulatory molecules, such as toll-like receptor (TLR) ligands, to drive cytokine release and antigen-presenting cell maturation, thereby enhancing the immunogenicity of co-delivered antigens [20]. Since such responses modulate the binding profile of vaccine-stimulated Abs, it is assumed that emulsion-based adjuvants drive VAERD by enhancing non-neutralizing HA2 stalk Abs.

In the current work, we investigated whether, apart from conventional emulsion-based adjuvants, VAERD can be triggered by the α-anomeric glycosphingolipid α-galactosylceramide (αGC). This compound, originally isolated from the sea sponge *Agelas mauritanius*, is presented by the non-classical major histocompatibility complex (MHC) class-I molecule CD1d on antigen-presenting cells to invariant natural killer T (iNKT) cells, activating them to secrete both pro- and anti-inflammatory cytokines [21,22,23,24,25]. The response is of such magnitude that αGC is considered among the strongest T-cell antigens discovered for any T lymphocyte [26]. Despite their low frequency in the body, iNKT cells activated with αGC have been shown to generate potent immunity against a wide range of co-delivered antigens in a fashion that resembles conventional CD4^+^ T-cell help [27,28,29,30]. This includes shaping CD4^+^ and CD8^+^ T-cell responses by licensing antigen-presenting cells (APCs) as well as by stimulating Ab affinity maturation and class switching through directly interacting with B cells presenting αGC on CD1d [31,32]. Since the discovery of αGC, numerous reports have shown that iNKT-cell responses can boost the immunogenicity of vaccines against a range of infectious diseases. Most of these studies have used the mouse IAV infection model and reported substantial enhancement in the magnitude and quality of influenza A virus (IAV) vaccine responses, including against heterologous challenge viruses (reviewed in [33,34]). While these results show the potential of exploiting the immunoregulatory activities of iNKT cells to improve vaccines, the question of whether this strategy risks triggering VAED remains uncertain.

In what follows, we show that αGC is capable of inducing VAERD with at least the same severity, in terms of clinical signs and lung pathology, as a conventional OIW adjuvant. However, instead of generating high concentrations of HA2-specific Abs, αGC-treated pigs accumulated high concentrations of IFN-γ-secreting cells, including in their lungs, indicating a different etiology of disease. These results serve as a note of caution that administering αGC to adjuvant WIV or peptide-based IAV vaccines could lead to potentially life-threatening pulmonary inflammation.

## 2. Material and Methods

### 2.1. Pigs

Twenty-seven mixed-breed pigs were obtained from Midwest Research Swine Inc. (Glencoe, MN, USA) and transported to the Large Animal Research Facility at Kansas State University (Manhattan, KS, USA). The animals arrived at four weeks of age and underwent a 5-day period of acclimatization to the research facility before the start of the study. A hemagglutination inhibition (HI) assay was used to confirm that the animals were seronegative for influenza H1 and H3 Abs as previously described [35]. The studies were in accordance with Kansas State University’s Institutional Animal Care and Use Committee (project number 4067) and Institutional Biosafety Committee (protocol number 1284). 

### 2.2. Viruses

The vaccine virus, a human-like δ1-cluster H1N2 A/swine/Minnesota02011/2008 (MN08) influenza A virus, was kindly provided by Dr. Amy Baker (USDA National Animal Diseases Center, Ames, IA, USA). Inactivation was performed by exposing the virus to UV light for 15 min, after which the virus preparation was passaged three times on Madin–Darby canine kidney (MDCK) cells to confirm inactivation. The challenge virus, H1N1 pandemic A/CA/04/2009 (CA04), was generated using a reverse genetics system as previously described [36]. Both viruses were propagated using MDCK cells and subjected to RT-qPCR and Sanger sequencing to confirm their identity.

### 2.3. Virus Titration

Virus isolation was performed in nasal swabs and bronchioalveolar lavage fluid (BALF) collected in Dulbecco’s modified Eagle’s medium (DMEM, Corning, Corning, NY, USA) with an antibiotic–antimycotic (100 U/mL penicillin G, 0.1 mg/mL streptomycin, and 0.25 µg/mL amphotericin B—Gibco Life Technologies, Carlsbad, CA, USA). Respiratory tissues were mechanically dissociated in infection media, filtered, and stored, as previously described [37].

Virus titers were calculated according to median tissue culture infectious dose (TCID_50_) after serial dilution and infection of MDCK cells. Briefly, virus stocks and samples collected during this study were serially diluted 10-fold in infection media with 1 μg/mL of L-(tosylamido-2-phenyl) ethyl chloromethyl ketone (TPCK)-treated trypsin (Worthington Biochemical Corporation, Lakewood, NJ, USA) and transferred to MDCK cells in 96-well microtiter plates. Cells were incubated at 37 °C with 5% CO_2_ for 48 h. For the tissue homogenate supernatant, samples were discarded 3 h post-inoculation, and fresh infection medium with TPCK-treated trypsin was added to MDCK cells for the remaining incubation time. After incubation, plates were fixed for 10 min at −20 °C with methanol and stained for influenza virus particles using a monoclonal Ab against influenza A virus nucleoprotein (NP) (HB65 hybridoma supernatant; ATCC, Manassas, VA, USA) followed by a polyclonal goat anti-mouse IgG conjugated to Alexa Fluor 488 (Invitrogen, Carlsbad, CA, USA) as the secondary Ab. Plates were read by indirect immunofluorescence using an EVOS fluorescence microscope. TCID_50_ values were calculated by the method of Reed and Muench [38] and expressed as TCID_50_/mL or TCID_50_/g.

### 2.4. Experimental Design

The 27 pigs were randomly assigned to one of five treatment groups. On 0 and 21 days post vaccination (d.p.v.), pigs were immunized with 2 mL of the designated vaccine or sham vaccinated with vehicle, as described in Table 1 and Supplementary Figure S1. For vaccine preparation, 80 HA units of inactivated MN08 were mixed with 100 μg/kg of αGC (Diagnocine LLC, Hackensack, NJ, USA), dissolved in dimethyl sulfoxide (DMSO; Sigma-Aldrich, St. Louis, MO, USA) as previously described [39], and/or 20% *v/v* of a commercial OIW adjuvant (Emulsigen-D; Phibro Animal Health Corporation, Omaha, NE, USA). Sham-vaccinated pigs received the same volume of virus-free MDCK cell supernatant as the vaccine vehicle, and 50 μL/kg of DMSO as the αGC vehicle. At 35 d.p.v., 3 sham-vaccinated and non-challenged (SVNCh) pigs were necropsied after they were sedated with tiletamine–zolazepam (Telazol^®^; 4.4 mg/kg of body weight) and xylazine (2.2 mg/kg), and they were humanely euthanized with pentobarbital sodium IV injections (150 mg/kg of body weight). At 37 d.p.v., the remaining 24 pigs were sedated and intratracheally (i.t.) inoculated with 3 × 10^6^ TCID_50_ CA04 in 2 mL of DMEM. Body temperature and clinical signs were recorded daily throughout the challenge period. The degree of respiratory distress was scored for each pig from 0 to 3 as follows: 0: normal breathing; 1: sneezing, coughing, and nasal discharge; 2: labored breathing; 3: cyanosis marked by bluish or purplish discoloration of mucous membranes. Individual pigs were scored for clinical disease according to activity (active, lethargic, depressed, refused to move), respiratory signs (sneezing, coughing, nasal discharge, labored breathing, cyanotic extremities), body temperature (rectal temperature (°C) <40.0, 40.1–40.5, 40.6–41.0, >41.1 or <37.7), and appetite (normal, reduced interest in treats, refused treats, sunken flanks, loss of body weight). Peripheral blood was collected from the jugular vein into heparin-coated or serum collection vacutainer tubes (BD Biosciences, San Jose, CA, USA) at 0, 21, 37 d.p.v., and 5 days post infection (d.p.i.) for flow cytometry and HI assays. Peripheral blood mononuclear cells (PBMC) were isolated at 0 and 5 d.p.i. for IFN-γ ELISpot assays. Nasal swabs were collected at 0, 1, 3, and 5 d.p.i. to determine viral shedding. At 5 d.p.i. (42 d.p.v.), the infected pigs were euthanized as described above. During necropsy, BALF was collected from the left lung as previously described. Lung tissue, dorsal cervical lymph nodes (DCLNs), and tracheobronchial lymph nodes (TBLNs) were collected into DMEM for isolation of immune cells and analysis by flow cytometry or IFN-γ ELISpot assays. The TBLN and the right middle lung lobe were collected in formalin for histopathological analysis. Sections of trachea, bronchi, and individual lung lobes were fresh frozen for virus titrations.

### 2.5. Tissue Processing for Single-Cell Isolation

Tissue collection and processing for single-cell isolation was performed as previously described [39,40]. Briefly, white blood cells (WBCs) were isolated for flow cytometry after lysis of red blood cells (RBCs) with an ammonium-chloride-based lysis buffer. Peripheral blood mononuclear cells (PBMCs) were isolated and frozen as previously described [37]. BALF cell pellets were used for flow cytometry after centrifugation for the collection of supernatants. Approximately 2 g of lung tissue from the cranial, middle, and caudal lobes was minced and incubated with 5 μg/mL of Liberase TL (Roche Diagnostics Deutschland GmbH, Mannheim, Germany) for 45 min at 37 °C. After digestion, the samples were centrifuged and the supernatant with Liberase was discarded. The cell pellet was resuspended with fresh DMEM and filtered through a 100 μm cell strainer. Lymph nodes were homogenized using disposable tissue grinders (Fisher Scientific, Pittsburgh, PA, USA). Single cells were filtered through a 100 μm cell strainer and RBCs lysed. Then, cells were resuspended in cold PBS with 2% FBS and counted using a Countess™ II FL Automated Cell Counter (Life Technologies) and 0.4% trypan blue to determine cell viability.

### 2.6. Flow Cytometry and Antibodies

Single-cell suspensions were stained for flow cytometry in V-bottomed 96-well plates as previously described [37]. Briefly, cells were first stained for viability with a DNA fixable dye (LIVE/DEAD™ Fixable Near-IR Dead Cell Stain Kit, Invitrogen), followed by Fc blocking with recombinant rat IgG (Sigma-Aldrich), and then stained with a cocktail of monoclonal Abs to determine immune cell populations (details in Supplementary Table S1). T- and NK-cell subsets were distinguished using Abs specific for CD3ε (BB23-8E6-8C8; BD Biosciences), CD8α (76-2-11; Southern Biotech), CD8β (PPT23; Bio-Rad, Hercules, CA, USA), CD4 (74-12-4; Southern Biotech, Birmingham, AL, USA), TCRδ (PGBL22A; WSU Monoclonal Antibody Center, Pullman WA, USA), CD16 (G7; BD Biosciences), and CD11b (M1/70; BioLegend, San Diego, CA, USA). iNKT cells were identified using unloaded and PBS57-loaded mouse CD1d tetramers provided by the National Institutes of Health Tetramer Core Facility. Macrophages, monocytes, granulocytes, and dendritic cells (DCs) were distinguished using Abs specific for CD16, CD14 (MIL2; Bio-Rad), CD163 (2A10/11; Bio-Rad), CD172a (74-22-15A; BD Biosciences), CD11b, and MHC class II (H42A; WSU Monoclonal Antibody Center) (Supplementary Table S1 and Supplementary Figure S2). After staining, cells were fixed using the BD Cytofix/Cytoperm kit (BD Biosciences) and acquired on a BD LSRFortessa™ X-20 flow cytometer with FACSDiva software (version 8.0.1, BD Biosciences). Fluorescence-minus-one controls were used to determine positive and negative populations. Data were analyzed using FlowJo software (version 10.7.0, Treestar, Palo Alto, CA, USA).

To determine the intracellular expression of IFN-γ in TBLN cells, single-cell suspensions were incubated with or without 2 μL/mL Cell Stimulation Cocktail from eBiosciences (San Diego, CA, USA) and 1 μL/mL of the protein transport inhibitor BD GolgiStop. After 5 h incubation, cells were surface stained with Abs against CD3ε (BB23-8E6-8C8; BD Biosciences), CD8α (76-2-11, ThermoFisher, Waltham, MA, USA), and CD4 (74-12-4, ThermoFisher) and fixed and permeabilized using the FOXP3 Transcription Factor Fixation/Permeabilization kit (eBiosciences). Next, cells were re-stained with an anti-IFN-γ Ab (P2G10; BD Biosciences). Approximately 100,000 live leukocytes were collected per sample and acquired on a Cytek Aurora spectral flow cytometer (Cytek Biosciences, Fremont, CA, USA). Fluorescence-minus-one controls were used to gate cytokine-producing cells.

### 2.7. Cytokine Enzyme-Linked Immunosorbent Assay (ELISA)

Cytokine levels in BALF were quantified by ELISA to detect IL-1β, IL-2, IL-4, IL-6, IL-8, IL-10, IL-12/IL-23 p40, IFN-α, IFN-β, IFN-γ, GM-CSF, TNF-α, and TGF-β1 according to the manufacturer’s instructions. DuoSet ELISA kits from R&D Systems (Minneapolis, MN, USA) were used for porcine IL-1β, IL-2, IL-4, IL-6, IL-8, IL-10, IL-12/IL-23 p40, IFN-γ, GM-CSF, and TNF-α. A human DuoSet ELISA kit was used for TGF-β1. For IFN-α, we used an in-house-generated ELISA with recombinant porcine IFN-α and two anti-pig IFN-α Abs (clones K9 and F17; R&D Systems). One Ab was biotinylated with a biotin conjugation kit from Bio-Rad. To detect IFN-β, we used the swine IFN-β Do-It-Yourself ELISA kit from KingFisher Biotech (Saint Paul, MN, USA).

### 2.8. Next-Generation Sequencing

Viral RNA was extracted from a 5 d.p.i. nasal swab using the QIAamp Viral RNA Mini kit (Qiagen, Germantown, MD, USA). The extracted RNA was subjected to multi-segment RT-PCR to amplify the full length of all eight RNA segments [41]. The quality of the amplicons was analyzed in agarose gel electrophoresis and DNA was purified using the AMPure XP magnetic-bead-based system (Beckman Coulter, Brea, CA, USA). The purified PCR amplicons were quantified using the broad-range quantification kit for the Qubit 4 Fluorometer (ThermoFisher) and diluted to 0.2 ng/µL. A total of 1 ng of diluted amplicons was used for sequencing library preparation using the Illumina Nextera XT library preparation kit (Illumina, San Diego, CA, USA) according to the manufacturer’s instructions. The libraries were sequenced on the Illumina NextSeq using 150 bp paired-end reads with a mid-output kit.

Reads were demultiplexed and parsed into individual sample files that were imported into CLC Genomic Workbench version 22.0.1 (Qiagen) for analysis. Reads were trimmed to remove primer sequences and filtered to remove short and low-quality reads. The reads were mapped to the consensus sequence obtained from the challenge material. Following read mapping, all samples were run through a low-frequency variant caller module within CLC Genomic Workbench with a frequency cutoff greater than 2%. Heatmaps for each segment of the genome were generated for variants using R Studio (version 2022.12.0+353).

After initial analysis, single-nucleotide variations (SNVs) with frequencies above 2% were used to determine the count of nonsynonymous changes. 

### 2.9. Epitope Mapping of Nonsynonymous Mutations

A list of identified B- and T-cell epitopes present within CA04 antigens was obtained from the Immune Epitope Database (IEDB) [42]. Nonsynonymous mutations identified through next-generation sequencing were filtered to identify mutations with amino acid substitutions located within known CA04 B- and T-cell epitopes. Epitope amino acid substitutions were further sorted to identify those present in more than one animal and those occurring in one or more study groups for additional analysis.

### 2.10. IFN-γ ELISpot Assay

Frozen PBMCs were thawed using the thawing cell program of a ThermoMixer C with the cryo thaw Smartblock adaptor (Eppendorf, Framingham, MA, USA). Cells were washed twice with warm Roswell Park Memorial Institute (RPMI) thawing media (RPMI 1640, 20% FBS, 1× antibiotic–antimycotic), resuspended in T-cell culture media [RPMI 1640, 10% FBS, 1× antibiotic-antimycotic, and 55 μM 2-mercaptoethanol (Gibco)], and counted using a Countess™ II FL Automated Cell Counter. PBMCs were resuspended at 5 × 10^6^ cells/mL in T-cell culture media and rested for 2 h in an incubator (37 °C, 5% CO_2_). PBMCs were plated at 2.5 × 10^5^ or 5 × 10^5^ cells per well in 96-well MultiScreen HTS plates (Millipore, Billerica, MA, USA) pre-coated with anti-IFN-γ (P2G10, BD Biosciences). Single cells isolated from enzyme-digested lungs were plated immediately after isolation at 5 × 10^5^ or 1 × 10^6^ live cells per well and then incubated at 37 °C for 48 h with 0.1 MOI of live MN08 or CA04 virus or virus-free MDCK supernatant. The plates were then developed using a biotin-conjugated anti-IFN-γ mAb (P2C11, BD Biosciences), streptavidin-horseradish peroxidase (HRP) (BD Biosciences), and 3-amino-9-ethylcarbazole (AEC) substrate (BD Biosciences), according to the manufacturer’s instructions. The number and intensity of spots in each well were read using an AID iSpot EliSpot FluoroSpot Reader with AID EliSpot Software Version 7.0 (Advanced Imaging Devices GmbH, Strassberg, Germany). The data are presented as the IFN-γ release per 10^5^ live cells.

### 2.11. Antibody Detection and Quantification

Influenza-specific Abs in serum were quantified by serum virus neutralization (SVN) and hemagglutination inhibition (HI) assays. For HI, serum samples were treated with receptor-destroying enzyme II (Denka Seiken, Tokyo, Japan), heat inactivated, and incubated with chicken RBC (Colorado Serum Company, Denver, CO, USA) to remove non-specific agglutinants. Then, samples were serial diluted two-fold and incubated with MN08 or CA04, followed by incubation with 0.5% washed chicken RBCs as previously described [43]. The highest sample dilution that inhibited virus-induced RBC hemagglutination is presented.

The serum virus neutralization (SVN) assay was performed as previously described [7]. Briefly, 5 d.p.i. serum was heat-inactivated at 56 °C for 30 min, followed by a 2-fold serial dilution. Virus was added to diluted serum samples and incubated at 37 °C for 60 min. Next, dilutions were transferred to 96-well plates with confluent MDCK cells for inoculation, and then incubated at 37 °C for 48 h. Plates were fixed and stained for influenza NP following the immunofluorescent Ab (IFA) protocol described in the virus titration section. The neutralizing Ab titer was the highest dilution of serum that prevented infectivity of MDCK cells detected by IFA.

### 2.12. Anti-HA2 ELISA

Recombinant chimeric cH6/1 HA protein with the H6 head domain from A/mallard/Sweden/81/2002 (H6N1) and H1 stalk domain from A/California/04/09 (H1N1) were used to coat 96-well clear-bottomed plates (Immulon 4HBX, ThermoFisher) for the detection of stalk-specific Abs in serum by ELISA as previously described [44]. Briefly, cH6/1 was diluted to 2 μg/mL in PBS, and 50 μL per well was incubated for 12–16 h at 4 °C to coat plates. Coating buffer was discarded, wells were washed four times with PBS containing 0.1% Tween 20 (PBS-T), and they were blocked with PBS + 0.05% Tween 20 + 3% milk for 2 h at room temperature. Blocking solution was discarded, wells were washed four times with PBS-T, and serum dilutions were added in duplicate and incubated for 1.5 h at room temperature. Serum dilutions started at 1:100 with 2-fold serial dilutions to 1:12,800 in PBS + 0.05% Tween 20 + 1% milk. After 1.5 h, samples were discarded, wells were washed four times with PBS-T, and peroxidase-labeled rabbit anti-pig IgG Ab (1:3000 dilution, Sigma-Aldrich) was added to detect serum Abs bound to the antigen, and incubated for 1 h at room temperature. Secondary Ab was discarded, wells were washed four times with PBS-T, and 100 μL O-phenylenediamine dihydrochloride (OPD) substrate (SigmaFast OPD, Sigma-Aldrich) developing solution was added to wells for 10 minutes. The reaction was stopped with 50 μL 3 M hydrochloric acid, and the OD was measured at 490 nm by a Synergy 4 plate reader (BioTek Agilent, Santa Clara, CA, USA). Blank wells were coated with protein and incubated with secondary Ab. The endpoint cutoff was determined as the average plus 3 times the standard deviation of the three SVNCh pigs.

### 2.13. Pathology and Histopathology

At necropsy, the lungs were assessed for the surface area with red and depressed areas (atelectasis) characteristic of IAV-induced pneumonia. The percentage of each lung lobe affected by pneumonia was visually estimated, which was used to calculate a score for individual pigs based on the relative proportion of each lung lobe to the total lung: 10% for the left and right cranial and middle lobes, 5% for the accessory lobe, and 27.5% for the right and left caudal lobes, for a total of 100% [45]. The right middle lung lobe, which usually had the highest lesion scores, was fixed in 10% neutral phosphate-buffered formalin and stained with hematoxylin and eosin. Two sections of lung were blindly scored for histopathological lesions. Each lung section was scored from 0 to 3 for 6 separate criteria typically associated with IAV infections in pigs according to a previously described rubric that evaluated (i) epithelial necrosis, attenuation, or disruption; (ii) airway exudate-necrosis/inflammation; (iii) percentage of airways with inflammation; (iv) peribronchiolar and perivascular lymphocytic inflammation; (v) alveolar exudate; (vi) alveolar septal inflammation [14]. The total sum of the scores was calculated and graphed for each pig. A second slide of lung tissue was mounted and processed for immunohistochemistry analysis of CD3-positive cells following standard methods [40]. 

### 2.14. Statistical Analysis

Data were analyzed and graphed using GraphPad Prism version 9.4.0 (GraphPad Software, San Diego, CA, USA). The Shapiro–Wilk test was used to test the normality of the data by determining whether the distribution of scores deviated from a comparable normal distribution. Data for body temperature changes, respiratory clinical signs’ area under the curve (AUC), macroscopic lung lesions per lobe, histopathological lesion scores, blood iNKT-cell concentrations, lung double-positive T-cell concentrations, BALF cytokine concentrations, nasal swab viral titers, MN08 HI titers, anti-HA2 ELISA AUC, IFN-γ ELISpot in the blood, and counts of SNV were normally distributed and evaluated using a one-way or two-way analysis of variance (ANOVA). Means were separated using Tukey’s multiple-comparisons test when a main effect or interaction term was determined to be significant (*p* < 0.05). Data for respiratory clinical signs’ scores, macroscopic lesions for the total lung, iNKT-cell frequency and count in BALF and tissues, viral titers in BALF and tissues, HI titers against CA04, SVN titers, anti-HA2 ELISA, and IFN-γ ELISpot in the lungs were not normally distributed and therefore analyzed using a nonparametric Kruskal–Wallis test and Dunn’s multiple-comparisons test. Survival curves for animal shedding were analyzed by the Mantel–Cox log-rank test.

## 3. Results

### 3.1. iNKT-Cell Activation with Heterologous IAV Challenge Induces VAERD

To determine whether immune responses elicited by iNKT cells are capable of inducing VAERD, four-week-old mixed-breed pigs were vaccinated, and then boosted i.m. with 80 HA units of UV-inactivated δ1-cluster H1N2 A/swine/Minnesota/02011/2008 (MN08) mixed with 100 μg/kg of αGC (group αGC). Two additional groups were vaccinated with MN08 and the oil-in-water adjuvant (OIW) Emulsigen-D and the combination of OIW and αGC (OIWαGC), respectively. Sixteen days after the booster, all three groups were intratracheally challenged with 3 × 10^6^ TCID_50_ of heterologous pandemic H1N1 A/California/04/2009 (CA04) virus. Additional control groups included sham-vaccinated and non-challenged pigs (SVNCh) and pigs that were sham-vaccinated and challenged with CA04 (SV) (Table 1, Supplementary Figure S1).

**Figure 1:**
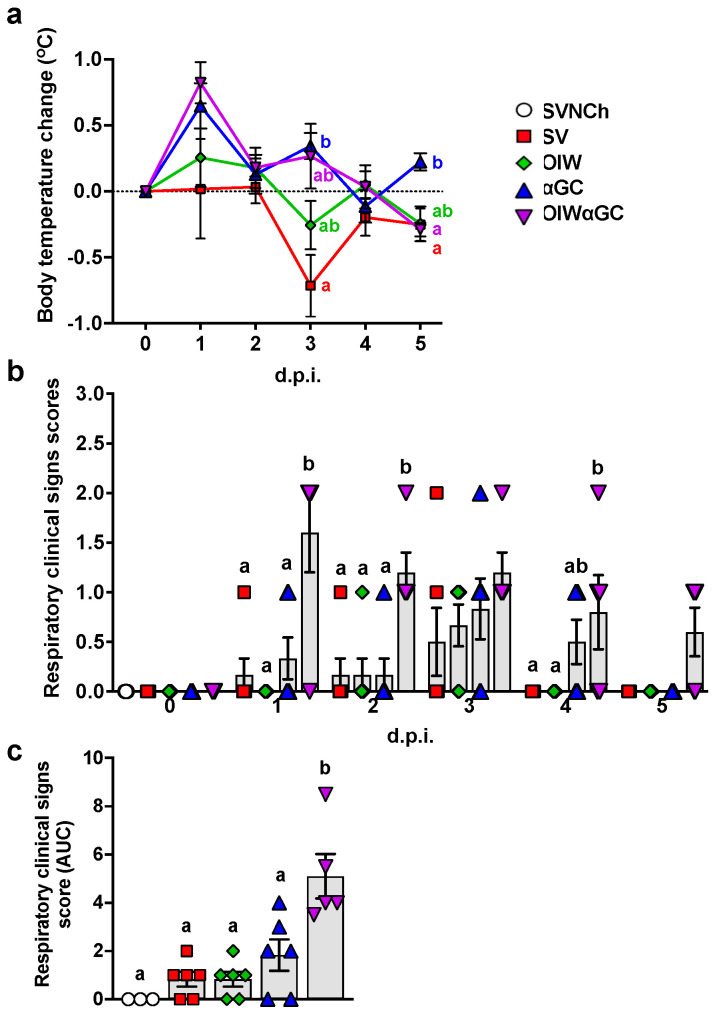
Clinical signs post-infection. (**a**) Change in body temperature during the challenge period based on body temperature at 0 d.p.i. (**b**,**c**) Respiratory clinical scores observed during the challenge period. Pigs were scored each day after infection according to the Materials and Methods (**b**). Area under the curve (AUC) calculated based on respiratory clinical scores over the entire challenge period (**c**). Differences between treatment groups were determined by Tukey’s (**a**,**c**) or Dunn’s (**b**) multiple-comparisons tests. A statistically significant difference between two groups is indicated by different letters. Data are presented as the mean ± SEM. Symbols represent treatment groups (**a**) or individual pigs (**b**,**c**). SVNCh—sham vaccinated and not challenged, SV—sham vaccinated and challenged with CA04, αGC—vaccinated with MN08 mixed with 100 μg/kg of αGC and challenged with CA04, OIW—vaccinated with MN08 and the oil-in-water adjuvant Emulsigen-D and challenged with CA04, OIWαGC—vaccinated with a combination of OIW and αGC and challenged with CA04.

After the second vaccination, 2/6 pigs that received the OIW and 5/6 of the pigs that received OIWαGC had redness and swelling at the injection site, which disappeared 3 to 4 days after vaccination. Following challenge, body temperature was elevated in αGC and OIWαGC pigs at 1 and 3 d.p.i. (Figure 1a). The OIW and SV groups did not develop pyrexia. Only αGC and OIWαGC pigs presented clinical signs typical of VAERD, including increased respiratory effort and coughing (Figure 1b,c). OIWαGC vaccinates showed the most severe signs of respiratory distress, with one pig meeting the criteria for early euthanasia at 1 d.p.i.

**Figure 2:**
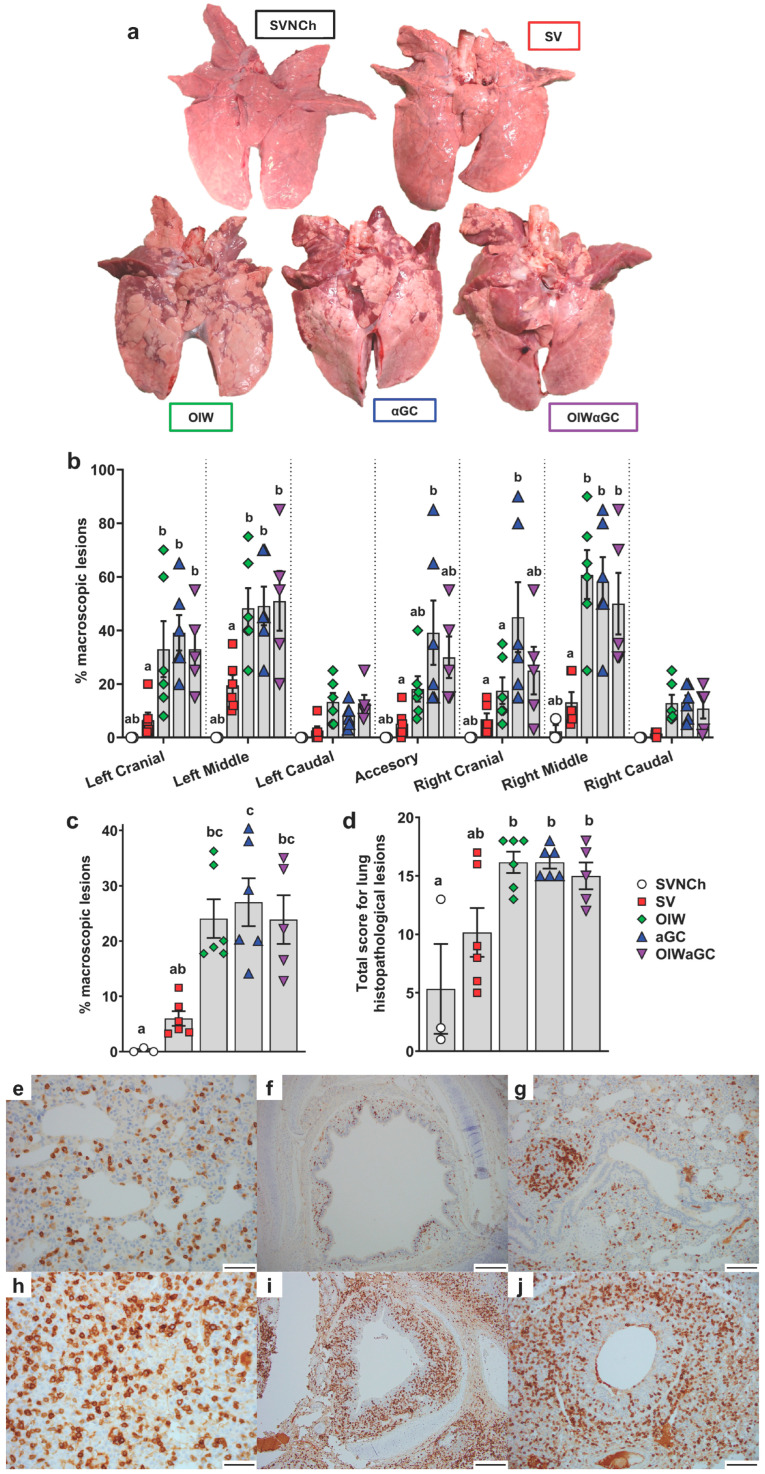
Macroscopic and microscopic lung lesion scores and CD3^+^ T-cell localization at 5 d.p.i. (**a**) Representative pictures of macroscopic lesions per group. (**b**,**c**) Macroscopic lesions assessed in (**b**) individual lung lobes and (**c**) total lungs according to the relative volume of each lobe. (**d**) Histopathology scores assessed by H&E staining according to the Materials and Methods. Differences between treatments were analyzed by Tukey’s (**b**,**d**) or Dunn’s (**c**) multiple-comparisons test. A statistically significant difference between two groups is indicated by different letters. Data are presented as the mean ± SEM. Symbols represent individual pigs. (**e**–**j**) Histopathology assessed by CD3 staining by IHC. (**e**–**g**) Images from SVNCh pigs of transverse sections of CD3-stained alveoli at 40× magnification (**e**), bronchus at 10x (**f**), and bronchiole with bronchus-associated lymphoid tissue at 20× (**g**). (**h**–**j**) Images from OIW pigs of transverse sections of CD3-stained alveoli at 40× with high-density intra-epithelial CD3^+^ T cells (**h**), bronchus at 10× with markedly thickened walls containing high-density aggregates of CD3^+^ T cells (**i**), and bronchiole at 20× showing high-density CD3^+^ cells in the bronchiolar wall within hyperplastic cuboidal bronchiolar epithelial cells (**j**). Scale bars indicate 50 μm (**e**,**h**), 200 μm (**f**,**i**), and 100 μm (**g**,**j**). SVNCh—sham vaccinated and not challenged, SV—sham vaccinated and challenged with CA04, αGC—vaccinated with MN08 mixed with 100 μg/kg of αGC and challenged with CA04, OIW—vaccinated with MN08 and the oil-in-water adjuvant Emulsigen-D and challenged with CA04, OIWαGC—vaccinated with a combination of OIW and αGC and challenged with CA04.

Necropsies were performed at 5 d.p.i., when immunopathology from VAERD is usually readily visible. All three vaccinated groups had a significantly higher percentage of lung surface area affected by atelectasis and pneumonia compared to unvaccinated challenged pigs (Figure 2a–c). Assessment of individual lung lobes revealed a tendency for more lesions in accessory and right cranial lung lobes of αGC pigs compared to the other adjuvanted groups (Figure 2b). The overall average macroscopic lesion scores per total lung were similar among OIW (24.1 ± 3.5%), αGC (27.0 ± 4.3%), and OIWαGC (26.2 ± 4.3%) vaccinates (Figure 2c), which were at least four times higher than in SV pigs (6.0 ± 1.3%). Histopathological assessment of microscopic lung pathology found that all three adjuvant groups had high scores for lesions characteristic of VAERD [14], especially alveolar exudate and inflammation (Figure 2d, Supplementary Table S2). Together, these results demonstrate that αGC induces VAERD with a severity that is similar in regard to clinical signs, respiratory distress, and lung pathology to VAERD induced by an OIW adjuvant.

Lung tissue was assessed by immunohistochemistry to evaluate the effects of vaccination and infection on T-cell recruitment and localization in the respiratory tract. In SVNCh pigs, only rare CD3^+^ T cells were observed in the alveolar epithelium and lamina propria of bronchioles (Figure 2e–g). Virus infection induced the accumulation of CD3^+^ cells in the bronchial and bronchiolar walls. All three groups of vaccinated pigs (OIW, αGC, OIWαGC) had similar high concentrations of CD3^+^ T cells that could be readily observed in adventitia and around bronchioles in lamina propria and surrounding bronchi (Figure 2h–j). The density of CD3^+^-cell infiltration was lower in pigs inoculated with virus alone (SV) compared to the vaccinated pigs. We observed that OIW and αGC pigs tended to have higher densities of CD3^+^ cells associated with hyperplastic bronchial and bronchiolar epithelium than OIWαGC pigs. 

### 3.2. Analysis of Immune Cells and Cytokines

**Figure 3:**
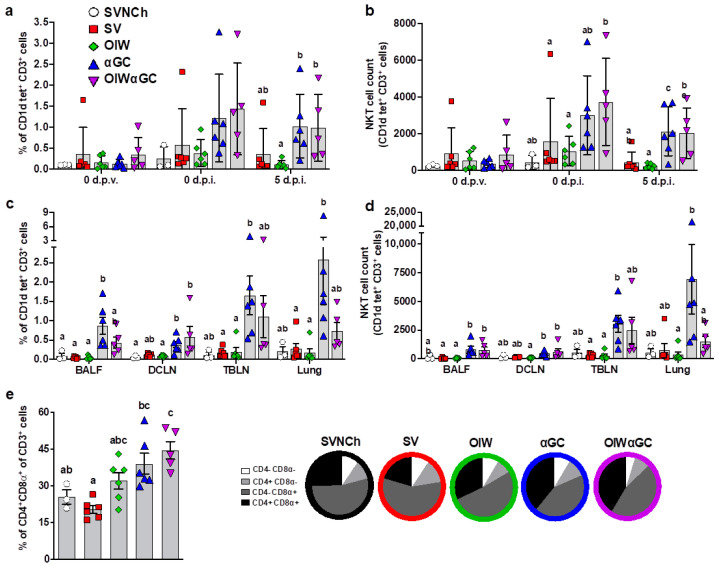
Immune cell characterization. (**a**,**b**) iNKT cells as a proportion of peripheral blood CD3^+^ lymphocytes (**a**) and the total number of iNKT cells in 10^6^ live cells at 0 d.p.v. and 0 and 5 d.p.i. (**b**). (**c**,**d**) iNKT cells in BALF, DCLN, TBLN, and lung tissue at 5 d.p.i. as a proportion of CD3^+^ lymphocytes (**c**) and the total number of iNKT cells (**d**). (**e**) Double-positive CD4^+^CD8α^+^ T cells as a proportion of total CD3^+^ cells in lung tissue at 5 d.p.i. Differences between treatment groups were determined by Tukey’s (**a**,**b**,**e**) or Dunn’s (**c**,**d**) multiple-comparisons test. A statistically significant difference between two groups is indicated by different letters. Data are presented as the mean ± SEM. Symbols represent individual pigs. SVNCh—sham vaccinated and not challenged, SV—sham vaccinated and challenged with CA04, αGC—vaccinated with MN08 mixed with 100 μg/kg of αGC and challenged with CA04, OIW—vaccinated with MN08 and the oil-in-water adjuvant Emulsigen-D and challenged with CA04, OIWαGC—vaccinated with a combination of OIW and αGC and challenged with CA04.

Flow cytometry was used to analyze immune cell populations, including αβ and γδ T-cell subsets, iNKT cells, NK cells, monocytes, macrophages, dendritic cells, and granulocytes within blood, bronchioalveolar lavage fluid (BALF), dorsal cervical lymph nodes (DCLNs), tracheobronchial lymph nodes (TBLNs), and lung tissue. Treatment groups vaccinated with αGC (αGC and OIWαGC) had higher concentrations of iNKT cells in peripheral blood at 37 d.p.v. (0 d.p.i.) and 5 d.p.i. (Figure 3a,b), and in BALF, DCLN, TBLN, and lung tissue at 5 d.p.i., compared to the other treatment groups (Figure 3c,d). αGC pigs tended to have higher iNKT-cell concentrations than OIWαGC pigs, especially in their lung tissue. We also observed that the lungs of vaccinated pigs, especially the αGC and OIWαGC groups, had higher frequencies of antigen-experienced CD4^+^CD8α^+^ T cells [46,47,48] compared to the unvaccinated pigs (Figure 3e). No significant differences in other cell types were observed among treatment groups.

**Figure 4:**
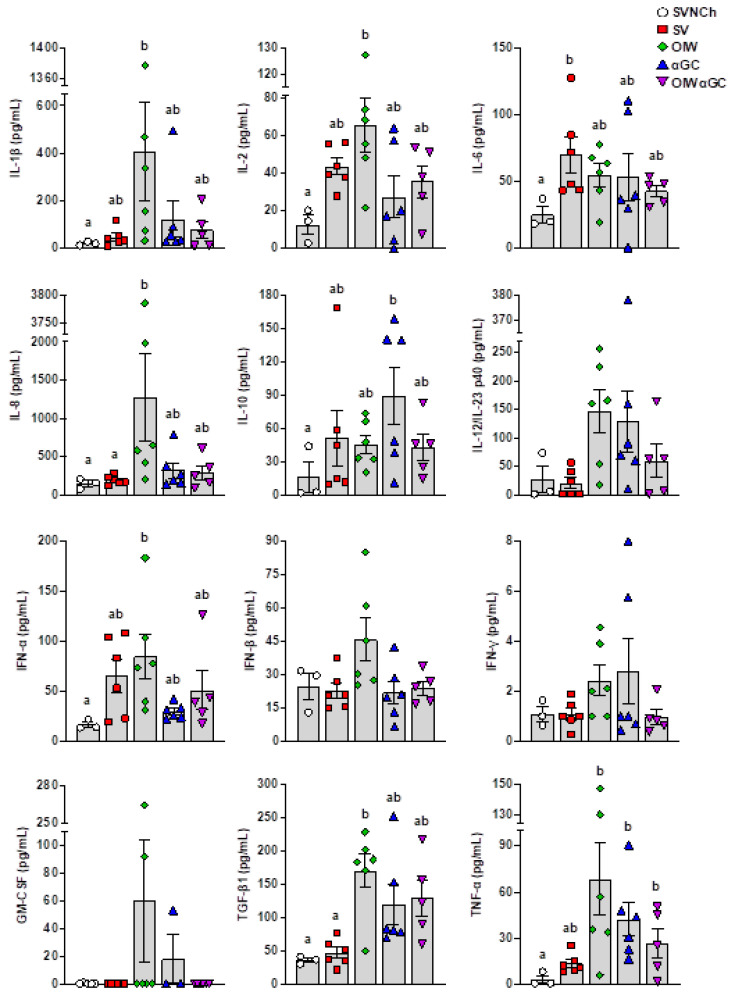
Cytokine concentrations in bronchiolar lavage fluid collected at 5 d.p.i. Differences between treatment groups were determined by Tukey’s multiple-comparisons test. A statistically significant difference between two groups is indicated by different letters. Data are presented as the mean ± SEM. Symbols represent individual pigs. SVNCh—sham vaccinated and not challenged, SV—sham vaccinated and challenged with CA04, αGC—vaccinated with MN08 mixed with 100 μg/kg of αGC and challenged with CA04, OIW—vaccinated with MN08 and the oil-in-water adjuvant Emulsigen-D and challenged with CA04, OIWαGC—vaccinated with a combination of OIW and αGC and challenged with CA04.

ELISAs were used to analyze IL-1β, IL-2, IL-4, IL-6, IL-8, IL-10, IL-12/IL-23 p40, IFN-α, IFN-β, IFN-γ, GM-CSF, TGF-β1, and TNF-α in BALF collected at euthanasia (Figure 4). IL-4 was not detected in any samples. We observed two patterns of cytokine expression. Pattern 1 was comprised of cytokines upregulated by all four infected groups compared to SVNCh pigs and included IL-2, IL-6, IL-10, and IFN-α. Among these, IFN-α and IL-2 were highest in OIW pigs while IL-10 was highest in αGC pigs. Pattern 2 consisted of cytokines that were increased in at least one vaccinated group compared to SV pigs (i.e., associated with VAERD) and included IL-1β, IL-8, IL-12/IL-23 p40, IFN-β, IFN-γ, GM-CSF, TGF-β1, and TNF-α. Among these, IL-1β, IL-8, IFN-β, and GM-CSF were upregulated in OIW pigs compared to the other vaccinated groups while IL-12/IL-23 p40, IFN-γ, TGF-β1, and TNF-α were also upregulated in OIW pigs and at least one of the αGC vaccinated groups. Interestingly, the combination of OIW and αGC did not additively or synergistically increase cytokine concentrations despite the high levels of pathology observed in OIWαGC pigs. Instead, most cytokines were present at lower concentrations in OIWαGC compared to the OIW group. This could mean that immune responses elicited by the OIW adjuvant and αGC are somewhat antagonistic or that OIWαGC cytokine responses had already subsided.

### 3.3. Virus Shedding and Replication

**Figure 5:**
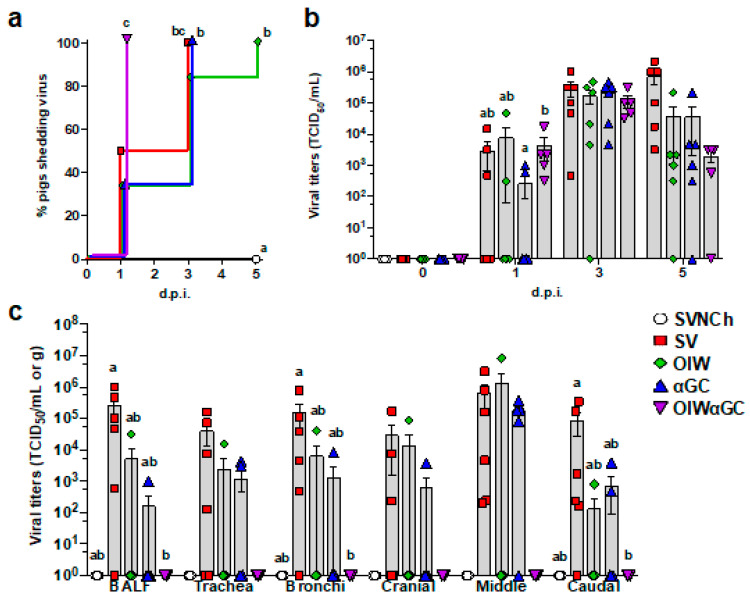
Viral titers in nasal swabs and respiratory tissues. (**a**) Percentage of pigs that have become positive for virus shedding in nasal swabs collected at 0, 1, 3, and 5 d.p.i. (**b**) Virus titers in nasal swabs after challenge. (**c**) Virus titers in BALF and homogenized respiratory tissues at 5 d.p.i. Data are presented as TCID_50_/mL for nasal swabs and BALF and TCID_50_/g for respiratory tissues. Differences between treatments were analyzed by the Mantel–Cox log-rank test (**a**), Tukey’s multiple-comparisons test (**b**), or Dunn’s multiple-comparisons test (**c**). A statistically significant difference between two groups is indicated by different letters. Data are presented as the mean ± SEM. Symbols represent treatment groups (**a**) or individual pigs (**b**,**c**). SVNCh—sham vaccinated and not challenged, SV—sham vaccinated and challenged with CA04, αGC—vaccinated with MN08 mixed with 100 μg/kg of αGC and challenged with CA04, OIW—vaccinated with MN08 and the oil-in-water adjuvant Emulsigen-D and challenged with CA04, OIWαGC—vaccinated with combination of OIW and αGC and challenged with CA04.

All OIWαGC pigs shed the virus by 1 d.p.i., whereas it took between 3 and 5 days for all pigs in the remaining challenge groups to shed the virus (Figure 5a). There was no significant difference in nasal swab virus titers among the infected groups. However, all three vaccinated groups tended to have lower virus concentrations than SV pigs at 5 d.p.i. (Figure 5b). Most SV pigs had high viral titers in BALF and respiratory tissues at 5 d.p.i. (Figure 5c). Vaccination with OIW and/or αGC reduced virus titers and the frequency of virus-positive pigs for BALF and tissue samples. In particular, OIWαGC pigs had almost no detectable virus.

### 3.4. Virus- and HA2 Stalk-Specific Antibody Responses

**Figure 6:**
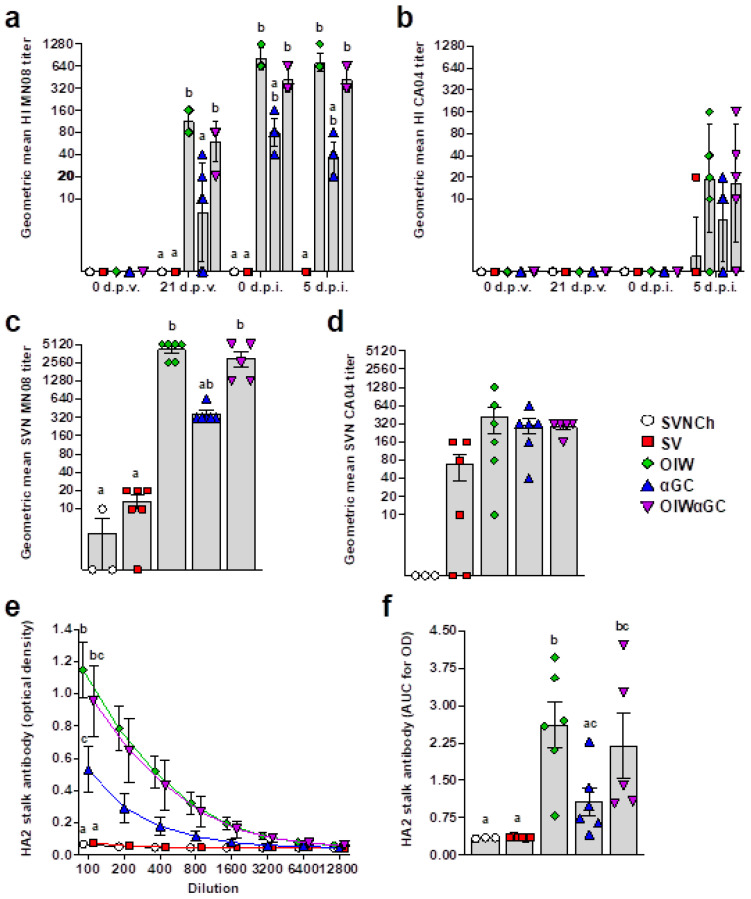
Virus-specific Ab titers. (**a**,**b**) Geometric mean of hemagglutination inhibition titers against H1N2 MN08 (a) and H1N1 CA04 (b). (**c**,**d**) Serum virus neutralization Ab titers against H1N2 MN08 (c) and H1N1 CA04 (d). (**e**,**f**) Ab titers against HA2 stalk of H1 influenza strains detected by ELISA at 5 d.p.i., represented as optical density by serum dilution (**e**) and area under the curve for optical density by dilution (**f**). Differences between treatments were analyzed using Tukey’s (**a**,**f**) or Dunn’s (**b**–**e**) multiple-comparisons test. A statistically significant difference between two groups is indicated by different letters. Data are presented as the geometric mean (**a**–**d**) or mean ± SEM (**e**,**f**). Symbols represent individual pigs (**a**–**d**,**f**) or treatment groups (**e**). SVNCh—sham vaccinated and not challenged, SV—sham vaccinated and challenged with CA04, αGC—vaccinated with MN08 mixed with 100 μg/kg of αGC and challenged with CA04, OIW—vaccinated with MN08 and the oil-in-water adjuvant Emulsigen-D and challenged with CA04, OIWαGC—vaccinated with a combination of OIW and αGC and challenged with CA04.

All vaccinated groups developed HI titers against the homologous MN08 vaccine strain (Figure 6a). However, the two groups that received OIW adjuvant (OIW and OIWαGC) presented higher HI titers than pigs vaccinated with αGC alone. None of the vaccinated groups had detectable HI titers against the heterologous CA04 challenge strain until 5 d.p.i., when very low titers were measured (Figure 6b). Serum virus-neutralizing Ab titers at 5 d.p.i. followed a similar pattern (Figure 6c,d). Abs against the HA2 region were quantified in serum collected at 5 d.p.i. by ELISA. All three vaccinated groups produced HA2-reactive Abs. However, both groups that received OIW (OIW and OIWαGC) had higher Ab concentrations than pigs that received αGC alone (Figure 6e,f). 

### 3.5. iNKT-Cell Activation Elicits High Concentrations of IFN-γ-Secreting Cells 

**Figure 7:**
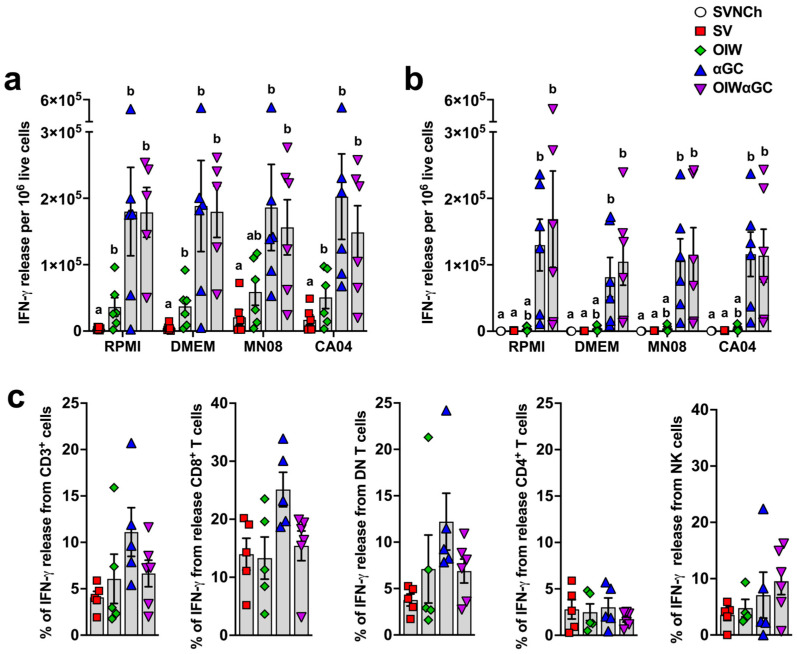
Cellular responses measured by IFN-γ production. (**a**,**b**) IFN-γ production by PBMC (**a**) or by lung leukocytes collected at 5 d.p.i. (**b**) after 48 h incubation with RPMI, DMEM, or 0.1 MOI of MN08 or CA04. Results represent IFN-γ release per 1 × 10^5^ live cells after subtracting unstimulated wells. (**c**) Proportion of T cells and NK cells positive for IFN-γ in TBLN. TBLN from 5 d.p.i. where incubated for 5 h with or without phorbol myristate acetate (PMA) and ionomycin, membrane labeled with Abs against CD3, CD4, and CD8α, fixed and permeabilized, and intracellularly stained for IFN-γ. Data are presented as the mean ± SEM. Symbols represent individual pigs. SVNCh—sham vaccinated and not challenged, SV—sham vaccinated and challenged with CA04, αGC—vaccinated with MN08 mixed with 100 μg/kg of αGC and challenged with CA04, OIW—vaccinated with MN08 and the oil-in-water adjuvant Emulsigen-D and challenged with CA04, OIWαGC—vaccinated with a combination of OIW and αGC and challenged with CA04.

Interferon-γ enzyme-linked immune absorbent spot (ELISpot) assays were performed to determine the concentration of vaccine and challenge virus-reactive PBMCs (Figure 7a) and lung cells (Figure 7b) at 5 d.p.i. Both groups that received αGC (αGC and OIWαGC) had cells producing very high concentrations of IFN-γ (~100-fold greater than pigs vaccinated with OIW alone) irrespective of whether or not they were restimulated with live virus. These results indicate that VAERD induced by αGC but not OIW adjuvant elicits immune cells that constitutively produce IFN-γ, both systemically and within the respiratory tract. 

To determine the identity of these cells, cryopreserved TBLN cells collected at necropsy were thawed and restimulated with PMA/ionomycin. They were then surface stained with Abs against CD3, CD4, and CD8α and intracellularly stained with anti-IFN-γ. CD8α^+^ T cells and CD4^−^CD8^−^ double negative (DN) T cells, which are primarily γδ T cells, from αGC and OIWαGC pigs tended to produce more IFN-γ than the other treatment groups, whereas CD4^+^ T cells and NK cells (CD3^−^CD8α^+^) produced similar amounts across treatments (Figure 7c). Thus, our ELISpot assay results in the αGC-treated groups were likely due to constitutive IFN-γ production from CD8 T cells and γδ T cells.

### 3.6. Vaccine-Induced Antibodies Correlate with Increased Genetic Diversity of IAV Genomes 

**Figure 8:**
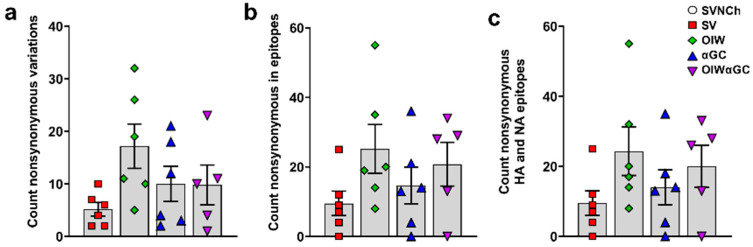
Nonsynonymous single-nucleotide variances detected in nasal swab samples collected at 5 d.p.i. (**a**) Total count of nonsynonymous SNVs per animal; (**b**) nonsynonymous SNVs located within epitopes of all CA04 proteins or (**c**) within HA and NA epitopes specifically. Differences between treatments were analyzed by Tukey’s multiple-comparisons test. Data are presented as the mean ± SEM. Symbols represent individual pigs. SVNCh—sham vaccinated and not challenged, SV—sham vaccinated and challenged with CA04, αGC—vaccinated with MN08 mixed with 100 μg/kg of αGC and challenged with CA04, OIW—vaccinated with MN08 and the oil-in-water adjuvant Emulsigen-D and challenged with CA04, OIWαGC—vaccinated with a combination of OIW and αGC and challenged with CA04.

To assess whether immune responses elicited by IAV infection and vaccination are associated with viral genome changes, we sequenced HA, NA, M, PB1, PB2, PA, NS, and NP cDNA amplicons obtained by RT-PCR from CA04 RNA recovered from the nasal swabs of each infected pig at 5 d.p.i. to identify SNVs (Supplementary File S1). Pigs in the three vaccinated groups had higher frequencies of nonsynonymous SNVs than SV pigs (Table 2 and Figure 8a). Moreover, OIW and OIWαGC pigs had more SNVs located within known T- and B-cell epitopes of CA04, but particularly within HA (Figure 8b) and NA (Figure 8c) epitopes [42] (Table 2). Collectively, these results demonstrate that VAERD-mediated immune responses drive the evolution of new virus variants. Moreover, VAERD induced by the OIW adjuvant elicited greater selective pressure than αGC, suggesting that vaccine-induced Abs, especially from the OIW adjuvanted pigs, contributed to the evolution of new virus variants following heterologous challenge. 

## 4. Discussion

Immunization with proteins and αGC activates iNKT cells to stimulate protein-specific humoral and cellular immunity. In mouse models, this adjuvant effect induces both primary and memory responses that can provide robust protection against cancer and a variety of pathogens, with Ab titers and T-cell levels that are usually as high as those elicited by conventional adjuvants (reviewed in [27]). Nevertheless, therapeutic iNKT-cell activation has had limited success for treating cancer or infectious disease in humans and non-human primates, possibly because most primates have lower numbers of iNKT cells than mice [49,50,51]. Moreover, attempts to therapeutically harness iNKT-cell responses in pigs, which also harbor relatively low concentrations of iNKT cells [39,52,53,54], have had mixed results. We previously showed that αGC increased the efficacy of a killed pdm2009 H1N1 IAV vaccine in pigs that were challenged with the homologous virus [40]. However, subsequent experiments found that αGC did not improve the ability of a live attenuated virus vaccine to provide cross-protection against a challenge with heterosubtypic viruses [37]. Moreover, efforts to replicate mouse studies, which found that treatment of active infections with αGC substantially reduces virus replication and disease, have had inconsistent outcomes in pigs, with one experiment finding a reduction in the viral load and two subsequent studies finding no effect [55,56,57]. Taken together, the immune responses elicited by αGC in pigs appear to be generally weaker than those observed in mice, resembling instead the more modest responses that this antigen elicits in most human and non-human primate studies. 

In contrast, the current VAERD study demonstrates that there are exceptions, where iNKT cells can have profound effects on immune responses in pigs. αGC elicited a robust presentation of VAERD associated with cells producing high concentrations of IFN-γ in both the lung tissue and systemically. These cells may underlie the αGC-induced form of VAERD since exuberant T-cell responses in the airway give rise to several pulmonary inflammatory disorders [58]. As regards other lung fluid cytokines, αGC-vaccinated pigs generally presented lower concentrations of pro-inflammatory cytokines, such as IL-1β, IL-8, IFN-β, and TNF-α, and higher concentrations of IL-10 compared to pigs vaccinated with OIW alone. Such results indicate significant differences in the mechanisms of VAERD induction by αGC versus emulsion-based OIW adjuvants, perhaps due to variation in antigen stability, release, and uptake or because of differences in how iNKT cells and emulsion-based adjuvants license APCs and induce the release of cytokines and chemokines from innate immune cells. Surprisingly, the two αGC-vaccinated pig groups had similar lung lavage fluid IFN-γ levels compared to the OIW group, which were all very low. This difference from the IFN-γ ELISpot results may be because IFN-γ produced in lung tissue is absorbed or degraded before it reaches the lung fluid or since our ELISA was not sensitive enough to quantify IFN-γ in lung fluid, which is highly diluted.

As regards humoral responses, the αGC group of pigs had significantly lower titers of vaccine-specific HI Abs, SNVs, and HA2-specific Abs than either of the two OIW-vaccinated groups. The reduced HA2 Ab levels in αGC pigs are interesting as it is currently assumed that emulsion-based adjuvants drive VAERD by enhancing non-neutralizing HA2 Abs [14]. However, αGC pigs had similar levels of clinical signs and lung pathology compared to the other vaccinated groups, indicating that virus-directed humoral immunity was not the driver of VAERD in these pigs. 

Consistent with previous VAERD studies [6,7,15,16,17,18,19], OIW and αGC pigs failed to generate HI titers against the heterologous challenge virus, nor did vaccination protect them from heterologous virus replication in the upper or lower airways compared to sham-vaccinated pigs. Combining αGC and OIW altered the dynamics of virus replication, with OIWαGC pigs shedding the virus earlier and clearing the virus in the lower respiratory tract sooner than the other vaccinated groups. Although VAERD induced by the combination of adjuvants did not increase lung pathology at 5 d.p.i., OIWαGC pigs had greater levels of respiratory distress and a more rapid onset of clinical signs compared to the other vaccinated groups. This could be due to heightened airway inflammation during the early stages of infection, which may explain why OIWαGC pigs cleared the virus from the lungs more rapidly than the other vaccinated groups.

Given that the vaccine-mediated immune responses that elicit VAERD are ineffective at inhibiting virus replication, it is not surprising that we observed greater genetic diversity in viruses recovered from the vaccinated compared to sham-vaccinated pigs. Although most SNPs were associated with individual pigs, diverse sequences of virus populations within individual hosts are an indication of selection and evolution of RNA viruses [59]. Mutations in structural and non-structural proteins were more prevalent in the two OIW-vaccinated groups, but especially HA and NA, which may be due to the high non-neutralizing HI titers and HA2 Abs produced by OIW-vaccinated pigs. Collectively, the higher incidence of nonsynonymous variants found in vaccinated compared to sham-vaccinated pigs indicates that immune responses elicited during VAERD drive the evolution of new IAV variants and that the OIW adjuvant stimulated greater levels of virus adaptation than αGC.

In conclusion, we have shown that immune responses originating from αGC activated iNKT cells can trigger VAERD in response to an influenza vaccination. In terms of clinical disease and lung pathology, αGC-induced VAERD was just as severe as VAERD induced by the conventional method of using an OIW adjuvant. However, iNKT-cell-mediated VAERD was associated with cells constitutively producing high concentrations of IFN-γ and significantly lower levels of non-neutralizing and anti-HA2 Abs than VAERD induced by the OIW. This is notable since vaccine-induced disease enhancement is usually attributed to Abs that increase viral infectivity, with cellular contributions presumed to play a secondary role. Accordingly, our results may indicate that distinct types of immune stimulation are capable of triggering VAERD if animals are exposed to the pertinent mismatched vaccine and challenge viruses or that anti-HA2 Abs may not be the primary driver for VAERD even in the OIW variant of the disease. In either event, our finding that iNKT-cell agonists can elicit VAERD-inducing immune activation raises concerns that should be taken into account if αGC is ever considered as an adjuvant for infectious disease and cancer vaccines in humans. 

## Figures and Tables

**Table 1:** Experiment setup.

Group	Experimental Group	Vaccine	αGC (μg/kg)	Emulsigen-D ^d^	Challenge Virus ^e^	*N*
1	SVNCh	Vehicle ^a^	Vehicle ^c^	0	None	3
2	SV	Vehicle	Vehicle	0	H1N1 CA04	6
3	OIW	MN08 ^b^	Vehicle	20%	H1N1 CA04	6
4	αGC	MN08	100	0	H1N1 CA04	6
5	OIWαGC	MN08	100	20%	H1N1 CA04	5 ^f^

^a^ Virus-free MDCK cell culture supernatant. ^b^ 80 HA units of UV-inactivated human-like δ1-cluster H1N2 A/swine/Minnesota/02011/2008 (MN08). ^c^ 50 μL/kg of dimethyl sulfoxide (DMSO) (the volume used to dissolve the 100 μg/kg dose of αGC used in groups 4 and 5). ^d^ Volume of oil-in-water adjuvant Emulsigen-D of total vaccine volume. ^e^ 3 × 10^6^ TCID_50_ H1N1 A/California/04/2009 administered intratracheally (i.t.) in 2 mL of DMEM. ^f^ One pig in group 5 was euthanized at 1 d.p.i. due to respiratory distress.

**Table 2:** Summary of nonsynonymous mutations overall and within B- and T-cell epitopes in virus recovered from nasal swabs.

Group	Experimental Group	Pig ID	Total Nonsynonymous Mutations	Number of Epitopes with Mutations ^a^			
2	SV	7785	6	9 HA			
		7791	10	25 HA			
		7800	4	7 HA			
		7804	2	4 HA			
		7809	7	12 HA			
		7813	2	-			
		Average	5.2				
3	OIW	7786	32	30 HA	2 NA	1 M1	2 NP
		7793	5	8 HA			
		7801	10	18 HA	2 NA		
		7805	19	17 HA	1 PB2	1 NEP/NS1	
		7810	26	50 HA	5 NA		
		7814	11	14 HA			
		Average	17.2				
4	αGC	7787	21	33 HA	2 NA	1 PB1	
		7795	2	4 HA			
		7802	18	15 HA	3 NA	3 NP	
		7806	4	13 HA			
		7811	12	14 HA			
		7815	3	-			
		Average	10				
5	OIWαGC	7789	7	23 HA	1 M1		
		7796	1	-			
		7803	10	33 HA	1 M1		
		7808	23	26 HA	2 M1	1 PB2	
		7812	11	25 HA	3 NA		
		7816	4	13 HA			
		Average	9.3				

^a^ Immune Epitope Database (IEDB) [42].

## Data Availability

All data produced and analyzed during this study are included in this manuscript. All relevant data are available from the authors.

## Supplementary Materials

The following supporting information can be downloaded at: https://www.mdpi.com/article/10.3390/vaccines12091068/s1, Figure S1. Experimental setup; Figure S2. Gating strategy to identify immune cell populations in blood, bronchioalveolar lavage fluid, lung, dorsal cervical lymph node and tracheobronchial lymph node; Table S1. Reagents used for flow cytometry analysis of surface markers; Table S2. Histopathological scores (average ± SEM) for individual criteria; File S1: Single-nucleotide variations in viral RNA extracted from a 5 d.p.i. nasal swabs.
