# Supplementary material for: Adjuvant Use of the Invariant-Natural-Killer-T-Cell Agonist α-Galactosylceramide Leads to Vaccine-Associated Enhanced Respiratory Disease in Influenza-Vaccinated Pigs
Source: Vaccines (Basel). 2024 Sep 18;12(9):1068. doi: 10.3390/vaccines12091068 (PMC11435877; doi:10.3390/vaccines12091068)
Supplement: Supplementary file 1 [file vaccines-12-01068-s001.zip › Supplementary figures and tables.pdf]

Supplementary Figure S1

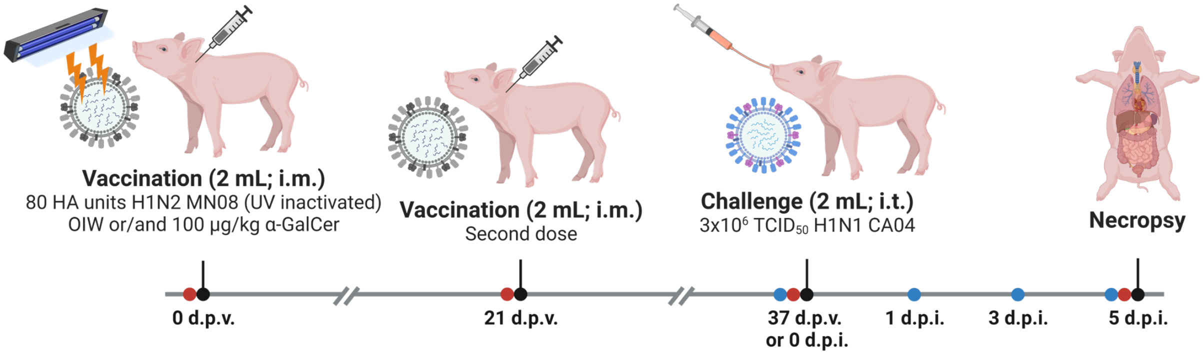

Supplementary Figure S1. Experimental setup. Pigs were vaccinated with 80 HA units of UV inactivated H1N2 MN08 in combination with commercial oil-in-water adjuvant, 100 µg/kg α-GalCer, or a combination of both adjuvants. The pigs received a second dose of the vaccine 21 days later and were challenged 16 days later (37 d.p.v. or 0 d.p.i.) with 3 x 10^6 $TCID_{50}$ of H1N1 CA04. Necropsies were performed at 5 d.p.i.. Timepoints for blood collections are represented by red circles, and nasal swab collections by blue circles. H1N2 MN08: human-like δ1-cluster H1N2 A/swine/Minnesota/02011/2008; H1N1 CA04: pandemic H1N1 A/California/04/2009; i.m.: intramuscular; i.t.: intratracheal; OIW: oil-in-water adjuvant; α-GalCer: α-galactosylceramide; d.p.v.: days post vaccination; d.p.i.: days post infection. Figure created with BioRender.com.

Supplementary Figure S2

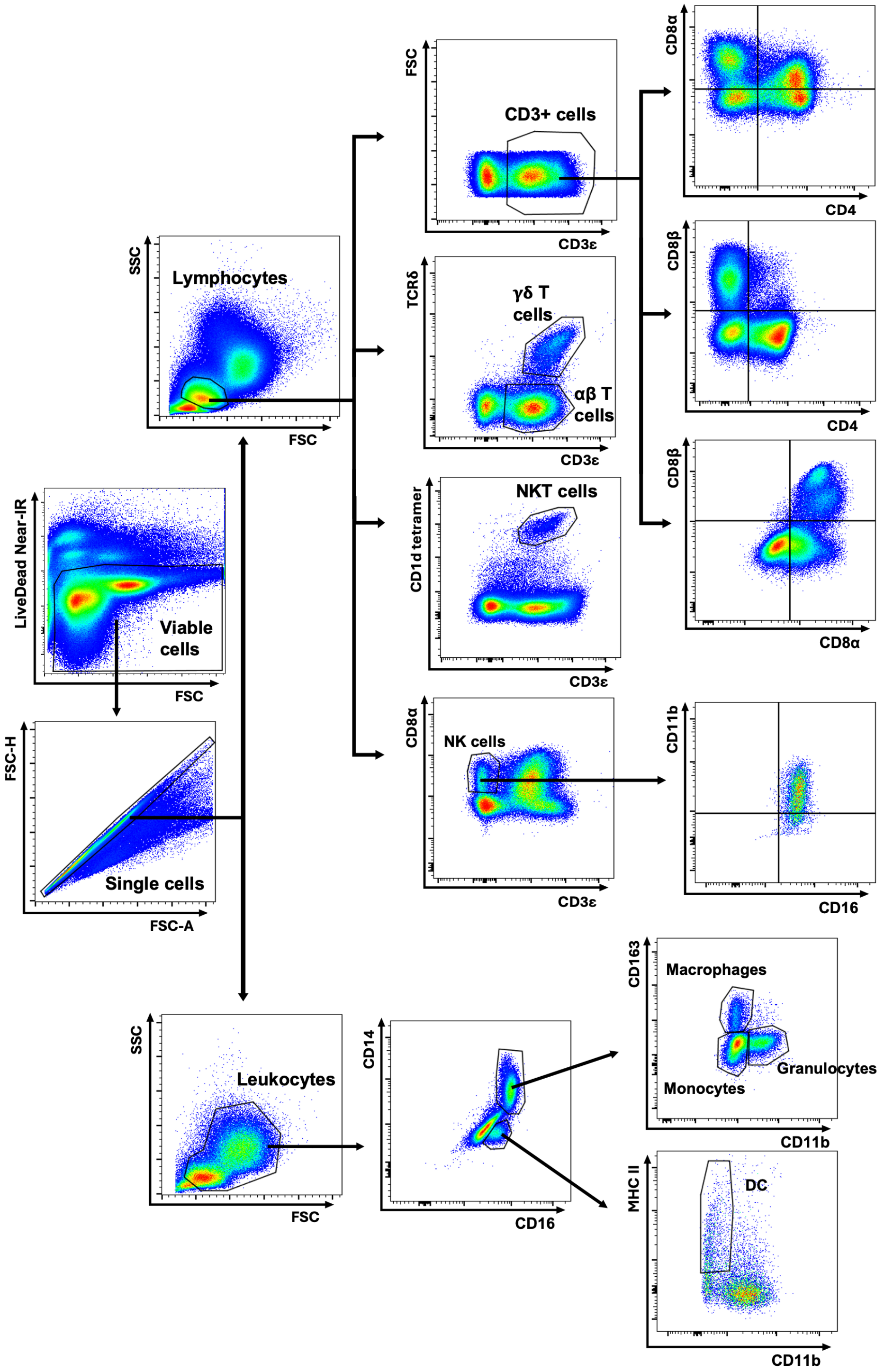

Supplementary Figure S2. Gating strategy to identify immune cell populations in blood, bronchioalveolar lavage fluid, lung, dorsal cervical lymph node and tracheobronchial lymph node. First, viable cells were selected, followed by single cells, then either lymphocytes or leukocytes were gated based on size. Within lymphocytes, total T cells, αβ and γδ T cells, iNKT cells, and NK cells populations were identified by the presence of CD3, TCRδ, CD4, CD8α, CD8β, and CD1d tetramer. Subsets of T cells were distinguished based on CD4, CD8α, and CD8β. For NK cells, subsets were defined based on CD11b and CD16 surface expression. Within leukocytes, we distinguished granulocytes, monocytes, macrophages, and dendritic cells. Granulocytes, monocytes and macrophages were positive for CD14 and CD16, then they were separated based on CD163 and CD11b markers. Dendritic cells (DC) were identified as $CD14^-$ $CD11b^-$ $MHCII^+$ cells. FSC, forward scatter; SSC, side scatter. One representative blood sample is shown.

Supplementary Table S1. Reagents used for flow cytometry analysis of surface markers.

Marker	Clone	Isotype	Source	Fluorochrome	Conjugation in house
CD3ε	BB23-8E6-8C8	Mouse IgG2aκ	BD Biosciences	PE-Cy7	N/A
NKT TCR	N/A	Mouse CD1d tetramer	NIH Tetramer Core	PE	N/A
CD4	74-12-4	Mouse IgG2bκ	Southern Biotech	AF647	AF647 (Invitrogen)
			BD Biosciences	PE	N/A
CD8α	76-2-11	Mouse IgG2aκ	Novus Biotech	AF405	N/A
			Southern Biotech	AF488	AF488 (Invitrogen)
CD8β	PPT23	Mouse IgG1	Bio-Rad	AF488	AF488 (Invitrogen)
TCRδ	PGBL22A	Mouse IgG1	WSU Mab Center	AF647	AF647 (Invitrogen)
CD16	G7	Mouse IgG1κ	BD Biosciences	Pacific Orange	Pacific Orange (Invitrogen)
CD11b	M1/70	Rat IgG2bκ	BioLegend	BV421	N/A
CD14	MIL2	Mouse IgG2b	Bio-Rad	AF488	AF488 (Invitrogen)
CD163	2A10/11	Mouse IgG1	Bio-Rad	PE	N/A
CD172α	74-22-15A	Mouse IgG2bκ	BD Biosciences	PerCP	PerCP LYNX Rapid (Bio-Rad)

CD: cluster of differentiation; Ig: immunoglobulin; PE: R-phycoerythrin; Cy: Cyanine; N/A: not applicable; NKT: natural killer T cells; TCR: T cell receptor; AF: Alexa Fluor; BV: Brilliant Violet; PerCP: Peridinin chlorophyll protein.

Supplementary Table S2. Histopathological scores (average ± SEM) for individual criteria.

Experimental group	Epithelial Necrosis/ attenuation/ disruption	Airway Exudate-Necrosis/ Inflammation	Percentage of airways with inflammation	Peribronchiolar and perivascular lymphocytic inflammation	Alveolar Exudate	Alveolar septal inflammation
SVNCh	0.67 ± 0.67	1.00 ± 1.00	0.67 ± 0.67	0.67 ± 0.67	1.00 ± 0.58	1.33 ± 0.33
SV	1.67 ± 0.42	2.17 ± 0.40	1.83 ± 0.40	1.00 ± 0.26	1.67 ± 0.42	1.83 ± 0.40
OIW	2.50 ± 0.34	2.83 ± 0.17	2.83 ± 0.17	2.33 ± 0.33	2.83 ± 0.17	2.83 ± 0.17
αGC	2.33 ± 0.21	2.83 ± 0.17	2.83 ± 0.17	2.33 ± 0.21	2.83 ± 0.17	3.00 ± 0.00
OIWαGC	2.33 ± 0.33	2.50 ± 0.22	2.67 ± 0.21	2.00 ± 0.26	2.83 ± 0.17	3.00 ± 0.00
